# Scattered Far-Field Sampling in Multi-Static Multi-Frequency Configuration

**DOI:** 10.3390/s21144724

**Published:** 2021-07-10

**Authors:** Maria Antonia Maisto, Mehdi Masoodi, Giovanni Leone, Raffaele Solimene, Rocco Pierri

**Affiliations:** Department of Engineering, University of Campania, 81031 Aversa, CE, Italy; mehdi.masoodi@unicampania.it (M.M.); giovanni.leone@unicampania.it (G.L.); raffaele.solimene@unicampania.it (R.S.); rocco.pierri@unicampania.it (R.P.)

**Keywords:** field sampling, inverse scattering, radar imaging

## Abstract

This paper deals with an inverse scattering problem under a linearized scattering model for a multi-static/multi-frequency configuration. The focus is on the determination of a sampling strategy that allows the reduction of the number of measurement points and frequencies and at the same time keeping the same achievable performance in the reconstructions as for full data acquisition. For the sake of simplicity, a 2D scalar geometry is addressed, and the scattered far-field data are collected. The relevant scattering operator exhibits a singular value spectrum that abruptly decays (i.e., a step-like behavior) beyond a certain index, which identifies the so-called number of degrees of freedom (NDF) of the problem. Accordingly, the sampling strategy is derived by looking for a discrete finite set of data points for which the arising semi-discrete scattering operator approximation can reproduce the most significant part of the singular spectrum, i.e., the singular values preceding the abrupt decay. To this end, the observation variables are suitably transformed so that Fourier-based arguments can be used. The arising sampling grid returns several data that is close to the NDF. Unfortunately, the resulting data points (in the angle-frequency domain) leading to a complicated measurement configuration which requires collecting the data at different spatial positions for each different frequency. To simplify the measurement configuration, a suboptimal sampling strategy is then proposed which, by an iterative procedure, enforces the sampling points to belong to a rectangular grid in the angle-frequency domain. As a result of this procedure, the overall data points (i.e., the couples angle-frequency) actually increase but the number of different angles and frequencies reduce and lead to a measurement configuration that is more practical to implement. A few numerical examples are included to check the proposed sampling scheme.

## 1. Introduction

Inverse scattering problems infer some features of an unknown object from its scattered field measurements once it has been illuminated by a known incident field [1]. This problem is of interest in several sensing and remote sensing applications, which range from nondestructive testing to geophysical prospecting, form microwave and ultrasonic medical imaging to buried-object detection.

As is well known, inverse scattering problems are very difficult to address since the non-linearity and the ill-posedness of the mathematical model must be dealt with. Usually, reconstruction procedures are cast as an optimization where the unknown is looked for as the one that minimizes a suitable misfit function. To this end, both deterministic and stochastic [2,3,4,5,6] approaches have been presented in the literature. However, these methods are in general computationally heavy and can suffer from reliability problems due to the occurrence of false solutions [7]. Accordingly, they are not convenient when it is required to diagnose an electrically large spatial region. In these cases, the scattering equations are usually linearized so that [8,9,10,11] computational inversion procedure can be exploited. For example, the Born approximation [8] is commonly adopted to achieve such a task. Although it is known that the Born approximation works for a very restricted class of targets (the ones addressed as weak scatters), it has been extensively shown in the literature that beyond its limits of validity, Born model reconstructions still allow to obtain qualitatively target reconstructions. This means that the target location and roughly its shape can be retrieved; fortunately, in many radar applications this is the main aim to pursue.

Besides linearization, in many applications, the far-field approximation can be exploited as well. The latter holds when the target and the measurement domain are very far from each other and consists of approximating the wavefront of the scattered field as a planar one. In this case, the scattering operator, linking the field and the target, enjoys a nice Fourier transformation structure [12].

In this framework, a very important issue concerns the measurement data collection [13,14,15]. Basically, data should be collected to trade-off the needs to keep the number of measurements as low as possible, which simplifies the measurement configuration and positively affects the acquisition time, and to avoid performance degradation while performing the target reconstruction. Accordingly, in this contribution we address such an issue by focusing on the case the scatted field is collected in far-field under a multi-static and multi-frequency configuration.

The problem of determining the set of data points can be more generally addressed as a sensors’ selection problem [16]. This, however, presents a combinatorial complexity which can be difficult to deal with in practice. For this reason, several different approximate methods have been proposed in the literature [17,18,19,20,21]. All these methods select the measurement points by iterative procedures that attempt to optimize some metrics related to the singular values of the scattering operator. Additionally, the number of measurement points to deploy is a priory fixed.

In this paper, our aim is to design a strategy to collect scattered field data which overcomes such difficulties by taking advantage from the mathematical properties of the scattering operator.

To this end we exploit the fact that the scattering operator is compact [22,23]. In particular, the far-field Green function, i.e., the kernel of the scattering operator, behaves similarly to an entire function of exponential type. This results in an abrupt decay of the singular values beyond a certain critical index, the so-called number of degrees of freedom (NDF) [23,24,25,26,27,28,29,30] of the scattered field. This singular value behavior, on one hand, is the result of the ill-posedness of the problem [31,32], which limits the achievable performance in the reconstructions. On the other hand, it entails that the range of the scattering operator, i.e., the set of all possible scattered fields, to be approximated by a finite-dimensional space, whose dimension depends on the NDF [22]. Accordingly, the measurement points can be determined as the ones that allow approximation of the subspace spanned by the “first” NDF left singular functions. It is known that such a subspace represents an *NDF-th width* of the range of the scattering operator [33], i.e., it is the subspace of dimension NDF that returns the lower approximation error. However, a sampling representation of the scattered field is more convenient since it is directly linked to the field samples.

Eventually, our problem can be recast as the determination of a sampling representation which is able to approximate the “first” NDF left singular functions. To this end, the sampling approach developed in [34] can be exploited along with the classical Whittaker–Kotelnikov–Shannon sampling theorem [35].

Such an approach has been already exploited in the framework of inverse source problems in [36,37,38], where near-field data were collected but only the spatial variable was considered. Instead, in this paper also the frequency diversity is taken into account. In particular, an optimal sampling strategy which minimizes the number of data in the angle-frequency domain is proposed for far-field data. This is done by suitably transforming the observation variables so that sampling approach mentioned above can be still exploited. However, although the proposed sampling scheme returns the number of data points close to the NDF, and hence equal to its minimal value, such a strategy leads to a complicated measurement configuration, which requires collecting the data at different angles for each different frequency. Hence, to simplify the measurement configuration, a suboptimal sampling strategy is introduced, which through an iterative procedure, enforces the sampling points to belong to a rectangular (not necessarily uniform) grid in the angle-frequency domain. In this way, the total number of scattered field data is of course increased (because they are not the optimum ones). However, the resulting measurement configuration is easier to implement and what is more the number of angle points at which to collect the data reduces.

## 2. Mathematical Formulation

**Figure 1:**
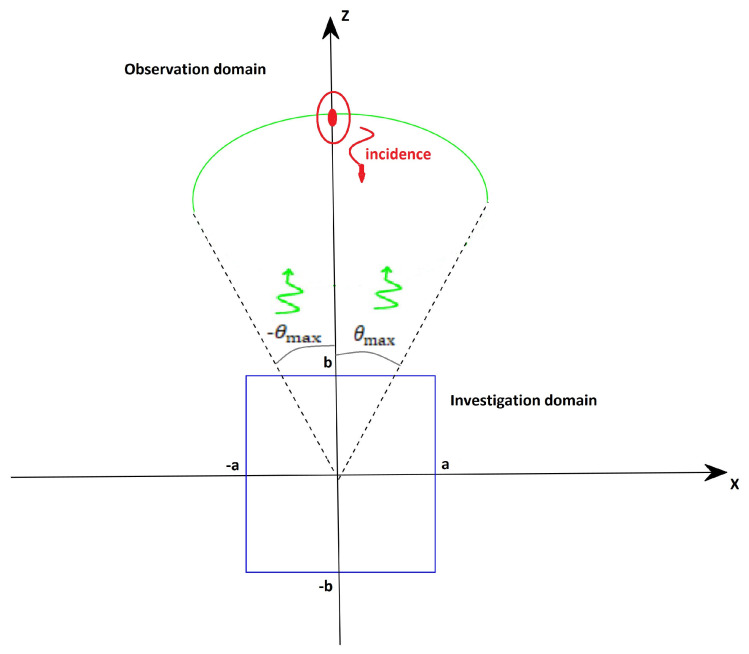
Considered geometry.

Consider the two-dimensional scalar scattering problem sketched in Figure 1, where invariance occurs along the *y* axis. The unknown scatterers are assumed to reside within a rectangular investigation domain ID=[−a,a]×[−b,b] located in free-space media. The scatterer region is illuminated by a plane wave with a fixed incidence angle $\theta_{i}$ and for different frequencies that result in propagation constant *k* ranging within $\Omega_{k}=\left[k_{min},k_{max}\right]$. The scattered field is collected with a multi-static, multi-frequency configuration under far-field conditions over an observation angular sector $\Omega_{o}=\left[-\theta_{\max}\;,\theta_{\max}\right]$, with $\theta_{\max}$ being the maximum observation angle. Accordingly, the scattering operator relating the contrast function χ describing the electromagnetic properties of the unknown targets and the only *y* component of scattered field *E* is written as (apart from some unessential scalar factors).

$$A:\chi \left(r\right)\in X=L^{2}\left(ID\right)\to$$

(1) $$\to E\left(k,\theta_{o}\right)=\int_{ID}\chi \left(r\right)e^{jk(\sin\left(\theta_{o}\right)-\sin\left(\theta_{i}\right)x}e^{jk(\cos\left(\theta_{o}\right)-\cos\left(\theta_{i}\right))z}dr\in Y=L^{2}\left(\Omega_{k}\times \Omega_{o}\right),$$

with $L^{2}\left(ID\right)$ and $L^{2}\left(\Omega_{o}\times \Omega_{k}\right)$ being the sets of square integrable functions supported over ID and $\Omega_{o}\times \Omega_{k}$, respectively, and r∈ID is the scatterer point. Please note that a scattering operator similar to (1) can be obtained by exploiting the Kirchhoff model to the scattering equation when strong scatterers are considered. Our aim is to design a strategy to collect the data on the rectangular domain $\Omega_{o}\times \Omega_{k}$ by following the same approach proposed in [36,37,38]. This consists of discretizing the data space so that the resulting discrete version of the eigenvalue problem associated with the left singular functions of A shares the first NDF eigenvalues of its continuous counterpart. In this way, according to the previous discussion, we are sure that the data space discretization allows approximating the “first” NDF left singular functions and, hence, the most important part of the range of the scattering operator.

Let ${\left\{u_{n},\sigma_{n},v_{n}\right\}}_{n=0}^{\infty }$ be the singular system of A, with $\sigma_{n}$ being the singular values and $u_{n}$ and $v_{n}$ the right and left singular functions that span the scatterer and the field spaces, respectively. It is well known that the $v_{n}$’s solve the following eigenvalue problem

(2) $$AA^{†}v_{n}=\sigma_{n}^{2}v_{n}$$

where $A^{†}$ is the adjoint of the scattering operator. Accordingly, to apply the sampling approach [34], we focus on $AA^{†}$ whose explicit expression, apart from an unessential constant, is

$$AA^{†}v_{n}=\int_{\Omega_{o}}\int_{\Omega_{k}}v_{n}\left(\theta_{o}^{\prime },k^{\prime }\right)\times$$

(3) $$\times \int_{ID}e^{jx(k\left[\sin\left(\theta_{o}\right)-\sin\left(\theta_{i}\right)\right]-k^{\prime }\left[\sin\left(\theta_{o}^{\prime }\right)-\sin\left(\theta_{i}\right)\right])}\;\;e^{jz(k\left[\cos\left(\theta_{o}\right)-\cos\left(\theta_{i}\right)\right]-k^{\prime }\left[\cos\left(\theta_{o}^{\prime }\right)-\cos\left(\theta_{i}\right]\right))}drdk^{\prime }d\theta_{o}^{\prime }$$

## 3. Optimal Sampling Strategy

To establish a sampling representation for $v_{n}$ and to devise the sampling scheme, the main idea it to recast the kernel function of $AA^{†}$ as a band-limited function. To this end, it is convenient to introduce the following couple of variables

(4) $$\omega \left(k,\theta_{o}\right)=k\left[\sin\left(\theta_{o}\right)-\sin\left(\theta_{i}\right)\right]\;\;\;\;\gamma \left(k,\theta_{o}\right)=k\left[\cos\left(\theta_{o}\right)-\cos\left(\theta_{i}\right)\right]$$

that are expressed in terms of $\left(k,\theta_{o}\right)$. Since the vectorial transformation $\Gamma :\left(k^{\prime },\theta_{o}^{\prime }\right)\to \left(\omega^{\prime },\gamma^{\prime }\right)$ with $\omega^{\prime }=\omega \left(k^{\prime },\theta_{o}^{\prime }\right)$, $\gamma^{\prime }=\gamma \left(k^{\prime },\theta_{o}^{\prime }\right)$ is injective and the corresponding Jacobian matrix is full rank, we can replace in (3) the integration in $\left(k^{\prime },\theta_{o}^{\prime }\right)$ with the integration in $\left(\omega^{\prime },\gamma^{\prime }\right)$, which yields

(5) $$AA^{†}v_{n}=\int_{\Omega }v_{n}\left(\omega^{\prime },\gamma^{\prime }\right)\frac{1}{k^{\prime }\left(\omega^{\prime },\gamma^{\prime }\right)}\int_{-a}^{a}\int_{-b}^{b}e^{jx(\omega -\omega^{\prime })}\;\;e^{jz(\gamma -\gamma^{\prime })}\;\;dx\;dz\;d\omega^{\prime }\;d\gamma^{\prime }$$

where $\Omega =\{\left(\omega^{\prime },\gamma^{\prime }\right):\left(\theta_{o}^{\prime },k^{\prime }\right)\in \Omega_{o}\times \Omega_{k}\}$. Basically, the rectangular domain in $\left(\theta_{o}^{\prime },k^{\prime }\right)$ (that is, $\Omega_{o}\times \Omega_{k}$) maps in $\left(\omega^{\prime },\gamma^{\prime }\right)$ as a sector of a disk. The kernel function in (5) is represented by the following integral

(6) $$ker\left(\omega ,\gamma ,\omega^{\prime },\gamma^{\prime }\right)=\frac{1}{k^{\prime }\left(\omega^{\prime },\gamma^{\prime }\right)}\int_{-a}^{a}\int_{-b}^{b}e^{jx(\omega -\omega^{\prime })}\;\;e^{jz(\gamma -\gamma^{\prime })}\;\;dx\;dz$$

By solving the integral, it becomes

(7) $$ker\left(\omega ,\gamma ,\omega^{\prime },\gamma^{\prime }\right)=\frac{1}{k^{\prime }\left(\omega^{\prime },\gamma^{\prime }\right)}H\left(\omega ,\gamma ,\omega^{\prime },\gamma^{\prime }\right)$$

with

(8) $$H\left(\omega ,\gamma ,\omega^{\prime },\gamma^{\prime }\right)=4ab\mathrm{sinc}\left[a\left(\omega -\omega^{\prime }\right)\right]\mathrm{sinc}\left[b\left(\gamma -\gamma^{\prime }\right)\right]$$

As can be seen, with respect to both (ω,γ) and $\left(\omega^{\prime },\gamma^{\prime }\right)$, $H(\omega ,\gamma ,\omega^{\prime },\gamma^{\prime })$ is the 2D inverse Fourier Transform of a rectangular window. In particular, if we focus only on the dependence in (ω,γ), the function *H* is a band-limited function with bandwidth equal to *a* and *b*, respectively. We are now in condition to build up the discrete counterpart of (5) by exploiting the procedure developed in [34]. More in detail, since $H(\omega ,\gamma ,\omega^{\prime },\gamma^{\prime })$ is a band-limited function, the standard sampling theorem can be exploited to obtain the following representation

(9) $$H\left(\omega ,\gamma ,\omega^{\prime },\gamma^{\prime }\right)=\sum_{m,l}H\left(\omega_{m},\gamma_{l},\omega^{\prime },\gamma^{\prime }\right)\mathrm{sinc}\left[a\left(\omega -\omega_{m}\right)\right]\mathrm{sinc}\left[b\left(\gamma -\gamma_{l}\right)\right]$$

with

(10) $$\left\{\begin{array}{c} \omega_{m}=m\pi /a\\ \gamma_{l}=l\pi /b \end{array}\right.$$

the sampling points of *H* with respect to ω and γ variables. Please note that as m∈Z and l∈Z with Z the set of integer numbers, (10) returns a rectangular sampling grid in ω and γ. By putting (9) in (5) we obtain

(11) $$AA^{†}v_{n}=\sum_{m,l}\left[\int_{\Omega }v_{n}\left(\omega^{\prime },\gamma^{\prime }\right)\frac{1}{k^{\prime }\left(\omega^{\prime },\gamma^{\prime }\right)}H\left(\omega_{m},\gamma_{l},\omega^{\prime },\gamma^{\prime }\right)d\omega^{\prime }\;d\gamma^{\prime }\right]\mathrm{sinc}\left[a\left(\omega -\omega_{m}\right)\right]\mathrm{sinc}\left[b\left(\gamma -\gamma_{l}\right)\right]$$

Equation (11) suggests that the eigenfunction $v_{n}$ can be expressed as

(12) $$v_{n}\left(\omega ,\gamma \right)=\sum_{m,l}v_{n}\left(\omega_{m},\gamma_{l}\right)\mathrm{sinc}\left[a\left(\omega -\omega_{m}\right)\right]\mathrm{sinc}\left[b\left(\gamma -\gamma_{l}\right)\right]$$

Equation (12) leads to two main consequences. On one hand, since the eigenfunctions $v_{n}$ span the closure of the Range of A, also the scattered field can be represented by the sampling series in (12). This means that according to (12), the field must be collected with a uniform step equal to π/a and π/b in the variables $\omega (k,\theta_{o})$ and $\gamma (k,\theta_{o})$. Because of the non-linear relationship between (ω,γ) and $\left(\theta_{o},k\right)$ (see (4)), the uniform rectangular grid in the ω−γ domain, described by (10), becomes non-uniform and of more general shape in the $\theta_{o}-k$ domain. The second implication of (12) is that it suggests how to build the discrete counterpart of the eigenvalue problem reported in (2). In fact, by substituting (12) within (11) and by evaluating the result at $\omega =\omega_{m}$ and $\gamma =\gamma_{l}$, one obtains

(13) $$\sigma_{n}^{2}{\underline{v}}_{n}=B{\underline{v}}_{n},$$

where ${\underline{v}}_{n}={\left\{v_{n}\left(\omega_{m},\gamma_{l}\right)\right\}}_{\alpha (m,l)=1}^{\infty }$ and α varies according to the way ${\underline{v}}_{n}$ is vectorized and $B=\{B_{\alpha ,\beta }\}$ with $B_{\alpha ,\beta }$ given by

(14) $$B_{\alpha (m,l),\beta (s,t)}=\int_{\Omega }\frac{1}{k^{\prime }\left(\omega^{\prime },\gamma^{\prime }\right)}H\left(\omega_{m},\gamma_{l},\omega^{\prime },\gamma^{\prime }\right)\mathrm{sinc}\left[a\left(\omega^{\prime }-\omega_{s}\right)\right]\mathrm{sinc}\left[b\left(\gamma^{\prime }-\gamma_{t}\right)\right]dk^{\prime }\;d\theta_{o}^{\prime }$$

Please note that the integer indexes *m*, *l* and *s* and *t* range over the two-dimensional sampling lattice involved by (11) and the matrix entry indexes α and β vary according to the way the vectorization of ${\underline{v}}_{n}$ is achieved.

It is worth remarking that B describes an infinite discrete problem. However, it is evident that, if $k^{\prime }\neq 0$ the more relevant contribution to the integral in (14) comes from the couples $\left(\omega_{s},\gamma_{t}\right)$ and $\left(\omega_{m},\gamma_{l}\right)$ which belong to Ω, in fact only for such points the sinc functions contribute with their main lobes.Accordingly, we can consider a truncated version of B, i.e., $B_{N}$ of size N×N, which takes into account only the samples falling within such a domain, possibly with a slight oversampling factor along ω and γ, say it ν, to control the truncation error. In this way, we are sure to approximate the first more significant singular values and moreover, the number *N* can be used as an estimation of the number of degrees of freedom NDF [23,34], i.e., the number of singular values preceding the abrupt decay. Please note that to truncate B to $B_{N}$ is equivalent to represent both the eigenfunctions $v_{n}$ and the scattered field as

(15) $$v_{n}\left(\omega ,\gamma \right)=\sum_{(\omega_{m},\gamma_{l})\in \Omega }v_{n}\left(\omega_{m},\gamma_{l}\right)\mathrm{sinc}\left[a\nu \left(\omega -\omega_{m}\right)\right]\mathrm{sinc}\left[b\nu \left(\gamma -\gamma_{l}\right)\right]$$

and

(16) $$E\left(\omega ,\gamma \right)=\sum_{(\omega_{m},\gamma_{l})\in \Omega }E\left(\omega_{m},\gamma_{l}\right)\mathrm{sinc}\left[a\nu \left(\omega -\omega_{m}\right)\right]\mathrm{sinc}\left[b\nu \left(\gamma -\gamma_{l}\right)\right]$$

**Figure 2:**
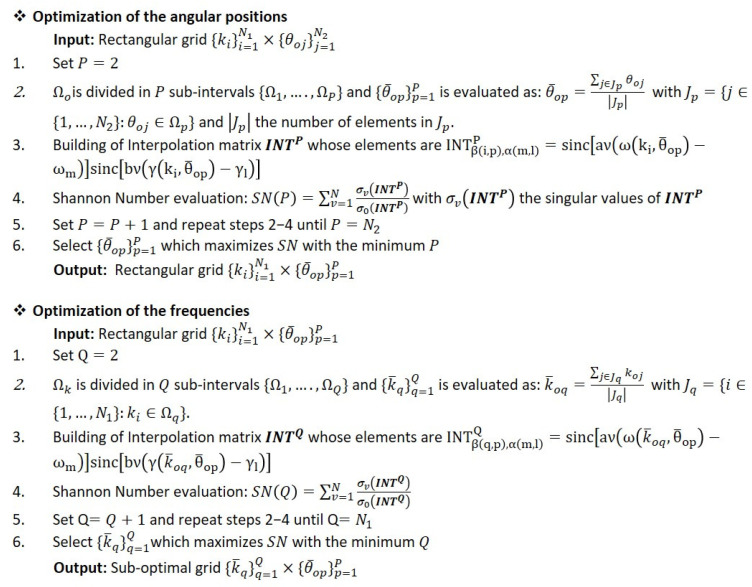
Suboptimal sampling strategy.

Equation (16) suggests not only how to collect the data but also how many samples *N* are required to approximate the more significant singular values of the continuous operator A. Summarizing, data should be collected with a uniform step in ω−γ which depends on investigation domain size. In this framework, among all possible sampling points in ω−γ only the ones fallen in Ω are relevant to represent the field. As mentioned before, since the non-linear relationship between the couples (ω,γ) and $\left(\theta_{o},k\right)$ reported in (4), the uniform rectangular grid in the ω−γ domain becomes non-uniform in the $\theta_{o}-k$ domain. This has a profound impact on the actual measurement configuration. Indeed, although the proposed sampling scheme returns several data which is close to NDF, and hence to the minimal number that in principle is required, such a strategy generally leads to a complicated measurement configuration that requires collecting the data at different angular positions for each different frequency. This of course is not too much practical and entails collecting the data over many different angles and frequencies, even if the total number of measurements ( the couples of angle-frequency) is minimized. To simplify the measurement configuration, a sub-optima sampling strategy, whose main steps are reported in Figure 2, is implemented which enforces the sampling points to belong to a rectangular grid in the $\theta_{o}-k$ domain. The latter scheme is suboptimal because it does require more data points than the previous one. However, those points are deployed over a rectangular grid and, what is more, the number of different angles and frequencies is reduced.

## 4. Suboptimal Sampling Strategy

Let ${\left\{k_{ml},\theta_{oml}\right\}}_{ml=1}^{N}$ be the optimal measurement grid obtained by the method explained in the previous section. Suppose that $N_{1}$ is the numbers of the different frequencies in the set of the optimal ones ${\left\{k_{ml}\right\}}_{ml=1}^{N}$ and $N_{2}$ the numbers of all different angles in ${\left\{\theta_{oml}\right\}}_{ml=1}^{N}$. From the latter, we build the rectangular grid ${\left\{k_{i}\right\}}_{i=1}^{N_{1}}\times {\left\{\theta_{oj}\right\}}_{j=1}^{N_{2}}$, which contains all the different frequencies and angles returned by the optimal sampling. However, this grid is much more populated than the previous one. Hence, it must contain redundant information that can be discarded. Accordingly, a rectangular sub-grid ${\left\{{\bar{k}}_{q}\right\}}_{q=1}^{Q}\times {\left\{{\bar{\theta }}_{op}\right\}}_{p=1}^{P}$ (with P,Q≤N) can be looked for. To achieve such a task, we implement an iterative procedure. More in details, starting from the rectangular grid ${\left\{k_{i}\right\}}_{i=1}^{N_{1}}\times {\left\{\theta_{oj}\right\}}_{j=1}^{N_{2}}$ we separately optimize (reduce) the angular positions and the frequencies. At the first, fixed ${\left\{k_{i}\right\}}_{i=1}^{N_{1}}$ we build the suboptimal vector ${\left\{{\bar{\theta }}_{op}\right\}}_{p=1}^{P}$ from ${\left\{\theta_{oj}\right\}}_{j=1}^{N_{2}}$ with the aim to minimize the spatial measurements numbers without degrading the singular values estimation. Next, once ${\left\{{\bar{\theta }}_{op}\right\}}_{p=1}^{P}$ has been estimated, the same procedure is applied to the frequencies to estimate ${\left\{{\bar{k}}_{q}\right\}}_{q=1}^{Q}$.

Figure 2 details the main steps of the suboptimal procedure. The first part concerns the iterative cycle for optimizing the angular positions and it has as input the rectangular grid ${\left\{k_{i}\right\}}_{i=1}^{N_{1}}\times {\left\{\theta_{oj}\right\}}_{j=1}^{N_{2}}$. The cycle starts by initializing the value of *P* at 2 and goes on by updating P=P+1 until it is equal to $N_{2}$. In each iteration, the interval $\Omega_{o}$ is divided in *P* sub-intervals $\left\{\Omega_{1},\ldots ,\Omega_{P}\right\}$ and for each *p*-th sub-interval ${\bar{\theta }}_{op}$ is evaluated as the mean value of the measurements ${\left\{\theta_{oj}\right\}}_{j=1}^{N_{2}}$ belonging to $\Omega_{p}$, explicitly

(17) $${\bar{\theta }}_{op}=\frac{\sum_{j\in J_{p}}\theta_{oj}}{|J_{p}|}$$

with $J_{p}=\left\{j\in \left\{1,\ldots ,N_{2}\right\}:\theta_{oj}\in \Omega_{p}\right\}$ and $|J_{p}|$ the number of elements in $J_{p}$. Please note that in the first iteration ${\left\{{\bar{\theta }}_{op}\right\}}_{p=1}^{P}$ contains only 2 elements and as the cycle goes on, the size of this vector increases. To ensure that the suboptimal angular grid ${\left\{{\bar{\theta }}_{op}\right\}}_{p=1}^{P}$ still allows for approximating the singular values of A, we consider the link between the samples of the eigenfunctions $v_{n}$ evaluated in $\left(\omega \left(k_{i},{\bar{\theta }}_{op}\right),\gamma \left(k_{i},{\bar{\theta }}_{op}\right)\right)$, denoted with ${\underline{\bar{v}}}_{n}^{P}$, and the samples in the optimal grid $\left(\omega_{m},\gamma_{l}\right)$, denoted as ${\underline{v}}_{n}$. Such a link can be found by evaluating (15) at $\omega =\omega (k_{i},{\bar{\theta }}_{op})$ and $\gamma =\gamma (k_{i},{\bar{\theta }}_{op}))$. The latter in matrix form becomes

(18) $${\underline{\bar{v}}}_{n}^{P}={\mathrm{INT}}^{P}{\underline{v}}_{n}$$

where explicitly ${\underline{\bar{v}}}_{n}^{P}=\{v_{n\beta (i,p)}=v_{n}\left(\omega \left(k_{i},{\bar{\theta }}_{op}\right),\gamma \left(k_{i},{\bar{\theta }}_{op}\right)\right\}$, ${\mathrm{INT}}^{P}=\left\{INT_{\beta (i,p),\alpha (m,l)}^{P}=\mathrm{sinc}\left[a\nu \left(\omega \left(k_{i},{\bar{\theta }}_{op}\right)-\omega_{m}\right)\right]\mathrm{sinc}\left[b\nu \left(\gamma \left(k_{i},{\bar{\theta }}_{op}\right)-\gamma_{l}\right)\right]\right\}$ and ${\underline{v}}_{n}=\left\{v_{n\alpha (m,l)}=v\left(\omega_{m},\gamma_{l}\right)\right\}$. It is evident that to approximate the optimal grid, we must be able to reconstruct with a good accuracy the vector ${\underline{v}}_{n}$ from ${\underline{\bar{v}}}_{n}^{P}$. In fact, only under this circumstance, the discrete equivalent eigenvalue problem (13) can be derived from the samples ${\underline{\bar{v}}}_{n}^{P}$. This means that the matrix ${\mathrm{INT}}^{P}$ must be well-conditioned. Please note that the size of the latter is $PN_{1}\times N$ and its mathematical properties depend on ${\left\{{\bar{\theta }}_{op}\right\}}_{p=1}^{P}$. If $PN_{1}\geq N$, it is expected that ${\mathrm{INT}}^{P}$ has *N* singular values whose value depends on the distribution of ${\left\{{\bar{\theta }}_{op}\right\}}_{p=1}^{P}$. Accordingly, the best angular positions are the minimal number of angles which maximize the *N* singular value level of ${\mathrm{INT}}^{P}$ and, hence, return a matrix ${\mathrm{INT}}^{P}$ with the best conditioning.

A measure of the conditioning of a matrix is given by the Shannon Number defined as

(19) $$SN\left({\mathrm{INT}}^{P}\right)=\sum_{v=1}^{N}\frac{\sigma_{v}\left({\mathrm{INT}}^{P}\right)}{\sigma_{1}\left({\mathrm{INT}}^{P}\right)}$$

where $\sigma_{v}\left({\mathrm{INT}}^{P}\right)$ are the singular values of ${\mathrm{INT}}^{P}$ sorted in descending order. Accordingly, we select the ${\left\{{\bar{\theta }}_{op}\right\}}_{p=1}^{P}$ which maximize such a number with the minimum *P*.

Next, once ${\left\{{\bar{\theta }}_{op}\right\}}_{p=1}^{P}$ has been estimated, the same procedure is applied to the frequency sampling to estimate ${\left\{{\bar{k}}_{q}\right\}}_{q=1}^{Q}$. Again, in order to select the best frequencies, we consider the *Shannon Number* of the interpolator ${\mathrm{INT}}^{Q}$ that now is defined as ${\mathrm{INT}}^{Q}=\left\{INT_{\beta (q,p),\alpha (m,l)}^{Q}=\mathrm{sinc}\left[a\nu \left(\omega \left({\bar{k}}_{q},{\bar{\theta }}_{op}\right)-\omega_{m}\right)\right]\mathrm{sinc}\left[b\nu \left(\gamma \left({\bar{k}}_{q},{\bar{\theta }}_{op}\right)-\gamma_{l}\right)\right]\right\}$ and whose dimension is PQ×N. By doing so, the discrete counterpart of the eigenvalue problem reported in (2) is given by

(20) $$\sigma_{n}^{2}{\underline{\bar{v}}}_{n}=B_{PQ}{\underline{\bar{v}}}_{n},$$

with $B_{PQ}=\left({\mathrm{INT}}^{Q}\right)\left(B_{N}\right){\left({\mathrm{INT}}^{Q}\right)}^{-1}$ with dimension PQ×PQ and ${\underline{\bar{v}}}_{n}=\left\{{\bar{v}}_{n\beta (q,p)}=v\left({\bar{k}}_{q},{\bar{\theta }}_{op}\right)\right\}$.

## 5. Numerical Example

In this section, we numerically check the previous theoretical findings. Assume that in (1) $ID=\left[-5\lambda_{max},5\lambda_{max}\right]\times \left[-5\lambda_{max},5\lambda_{max}\right]$, $\Omega_{o}=\left[-\frac{\pi }{3},\frac{\pi }{3}\right]rad$ and $\Omega_{k}=\left[k_{0},2k_{0}\right]$ with $k_{0}=\frac{2\pi }{\lambda_{max}}$ and $\lambda_{max}=1m$ the wavelength at minimum frequency.

**Figure 3:**
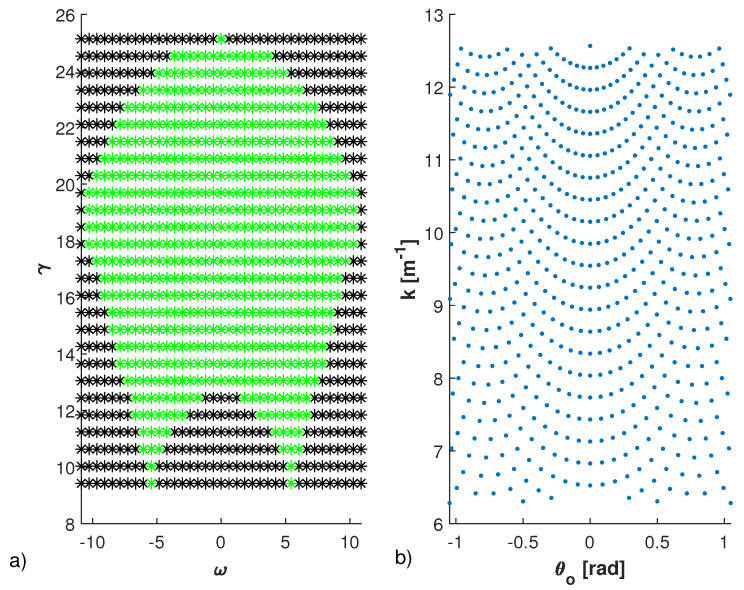
Sampling points obtained by collecting the field with uniform step equal to $\frac{\pi }{\nu a}$ and $\frac{\pi }{\nu b}$ with ν=1.05 in the variables $\omega (k,\theta_{o})$ and $\gamma (k,\theta_{o})$. In panel (**a**), the points are shown in ω−γ domain, while in panel (**b**) in $k-\theta_{o}$ domain. In particular, in panel (**a**) the black points are supported on a rectangular domain, while the ones that belong to Ω are shown in green color. In panel (**b**) only the N=619 points in $k-\theta_{o}$ domain that correspond to the green ones are shown. The parameters are $ID=\left[-5\lambda_{max},5\lambda_{max}\right]\times \left[-5\lambda_{max},5\lambda_{max}\right]$, $\Omega_{o}=\left[-\frac{\pi }{3},\frac{\pi }{3}\right]rad$ and $\Omega_{k}=\left[k_{0},2k_{0}\right]$ with $k_{0}=\frac{2\pi }{\lambda_{max}}$ and $\lambda_{max}=1m$ the wavelength at minimum frequency.

We first verify if the optimal and suboptimal sampling schemes approximate the eigenvalues of $AA^{†}$. In Figure 3, the optimal sampling grid ${\left\{k_{ml},\theta_{oml}\right\}}_{ml=1}^{N}$, corresponding only to the points of the rectangular grid in the ω - γ domain belonging to Ω (green points), is shown.

The optimal grid returns several points close to the NDF, equal to 619 in this case. As expected, the points are arranged in a non-uniform and non-rectangular way in the $\theta_{o}-k$ domain. This means that although the proposed optimal sampling scheme minimizes the total number of data, the number of different frequencies $N_{1}=599$ and of different angles $N_{2}=320$, corresponding to the 619 measurements, is rally very high.

**Figure 4:**
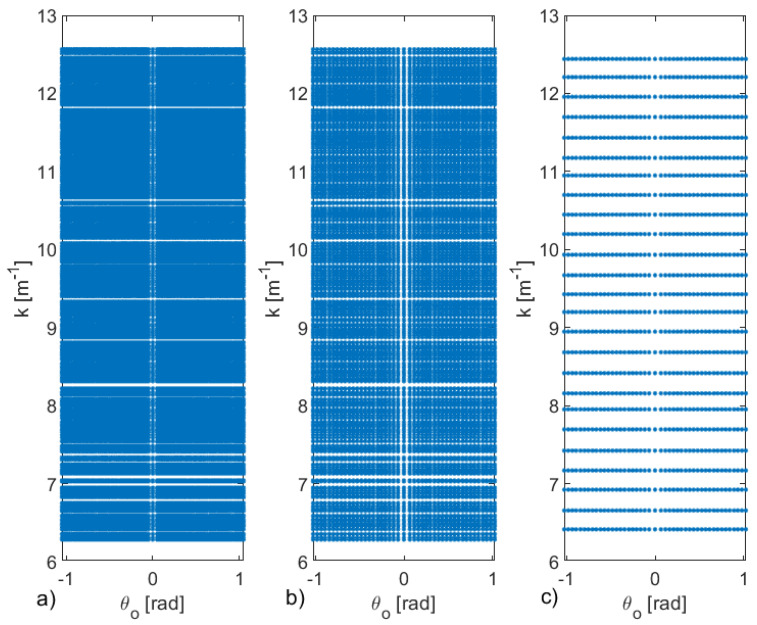
Panel (**a**) shows the rectangular grid ${\left\{k_{i}\right\}}_{i=1}^{N_{1}}\times {\left\{\theta_{oj}\right\}}_{j=1}^{N_{2}}$ built from the optimal grid ${\left\{k_{ml},\theta_{oml}\right\}}_{ml=1}^{N}$ (see panel (**a**) of Figure 4) with $N_{1}=599$ and $N_{2}=320$. The panels (**b**,**c**) refer to outputs of the two optimization steps of angular positions and of the frequencies in the proposed iterative suboptimal strategy, respectively. The procedure allows the estimation of the suboptimal grid ${\left\{{\bar{k}}_{q}\right\}}_{q=1}^{Q}\times {\left\{{\bar{\theta }}_{op}\right\}}_{p=1}^{P}$ with Q=25 and P=45. The parameters are the same of Figure 3.

To exploit the suboptimal approach, as the first step, a rectangular grid ${\left\{k_{i}\right\}}_{i=1}^{N_{1}}\times {\left\{\theta_{oj}\right\}}_{j=1}^{N_{2}}$ is built from the optimal grid ${\left\{k_{ml},\theta_{oml}\right\}}_{ml=1}^{N}$ (see panel (a) of Figure 4). As expected, the corresponding grid is much denser than the optimal one, but it is rectangular.

**Figure 5:**
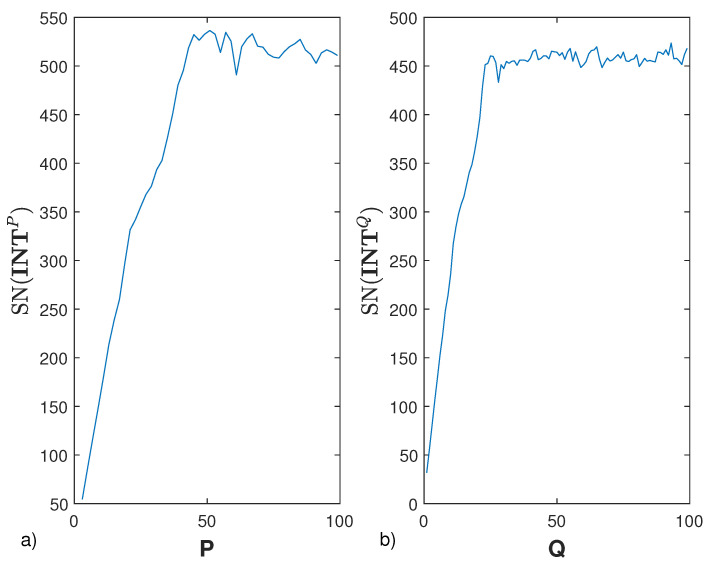
The Shannon Number of ${\mathrm{INT}}^{P}$ (panel 9**a**)) and ${\mathrm{INT}}^{Q}$ (panel (**b**)) in terms of *P* and *Q*. The parameters are the same of Figure 3.

Next, the two procedures to optimize the angles and the frequency are run. The output of the first procedure, the one concerning the optimization of the angles, is shown in panel (b) of Figure 4. In particular, this first stage returns a rectangular gird ${\left\{k_{i}\right\}}_{i=1}^{N_{1}}\times {\left\{{\bar{\theta }}_{op}\right\}}_{p=1}^{P}$, with P=45. The second stage, concerning the optimization of the frequencies is shown in panel (c) of the same figure, where the final grid ${\left\{{\bar{k}}_{q}\right\}}_{q=1}^{Q}\times {\left\{{\bar{\theta }}_{op}\right\}}_{p=1}^{P}$, with Q=25 is reported. As can be seen, although (as expected) the total number of data is increased to PQ=1125, the number of different angles and frequencies to be used is dramatically reduced to only 45 and 25, respectively. Figure 5 shows the behaviors of $SN({\mathrm{INT}}^{P})$ and $SN({\mathrm{INT}}^{Q})$ in terms of *P* and *Q*, which reach the maximum the first time at P=45 and Q=25, respectively.

**Figure 6:**
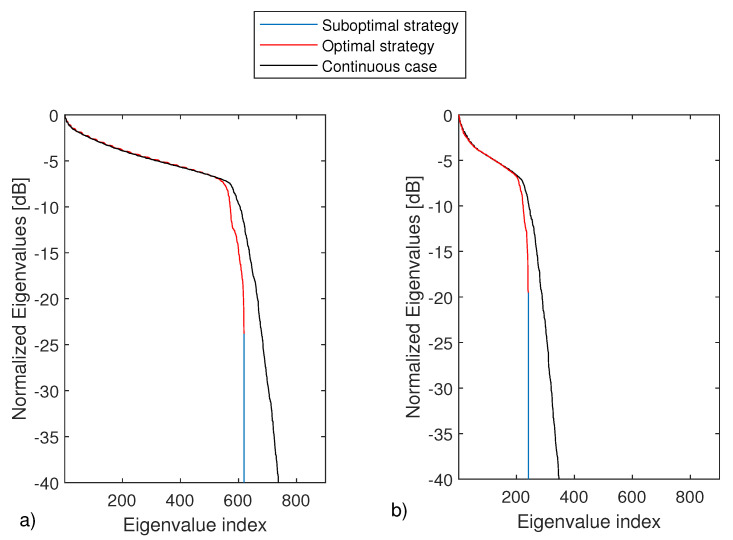
Eigenvalues of $B_{PQ}$ (blue lines), $B_{N}$ (red lines) and $AA^{†}$ (black lines). In panel (**a**), the parameters are the same of Figure 3. In panel (**b**), $ID=\left[-3\lambda_{max},3\lambda_{max}\right]\times \left[-6\lambda_{max},6\lambda_{max}\right]$, $\Omega_{o}=\left[-\frac{\pi }{2},\frac{\pi }{2}\right]rad$ and $\Omega_{k}=\left[k_{0},\frac{3}{2}k_{0}\right]$, respectively.

In panel (a) of Figure 6 the eigenvalues of $B_{PQ}$, $B_{N}$ and $AA^{†}$ are shown. Please note that the eigenvalues of $AA^{†}$ represents the benchmark against to compare the discrete approximations coming from the sampling schemes. More in details, those eigenvalues have been obtained by sampling the frequencies and the angles very densely so to obtain a good approximation of the continuous operator.

As can be seen, the eigenvalues returned by the two sampling schemes (the optimal and the suboptimal one) practically overlap and very well approximate the more significant eigenvalues of the continuous operator.

**Figure 7:**
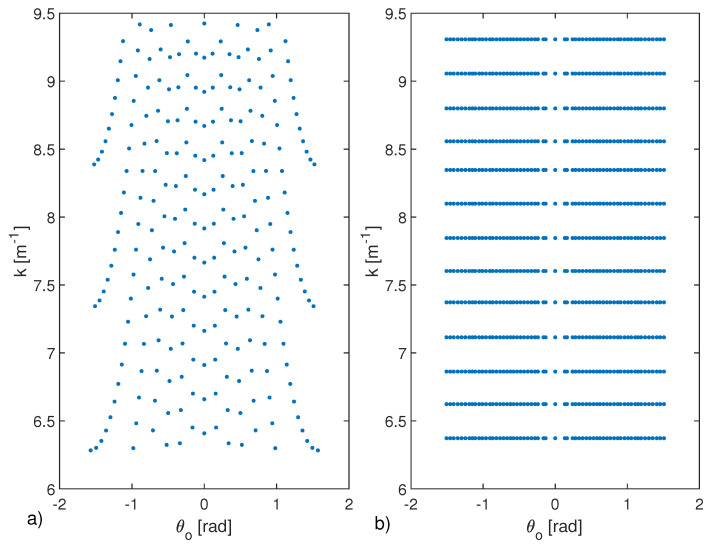
Optimal (panel (**a**)) and suboptimal (panel (**b**)) sampling grid. The parameters are the same as panel (**b**) of Figure 6. In this case, N=241, while P=61 and Q=13.

Panels (b) of Figure 6 refers to a different example, with $ID=\left[-3\lambda_{max},3\lambda_{max}\right]\times \left[-6\lambda_{max},6\lambda_{max}\right]$, $\Omega_{o}=\left[-\frac{\pi }{2},\frac{\pi }{2}\right]rad$ and $\Omega_{k}=\left[k_{0},\frac{3}{2}k_{0}\right]$. In Figure 7 shows the corresponding optimal (panel a)) and suboptimal (panel b)). Additionally, in these cases, the same conclusions as above can be drawn.

Finally, we end this section by showing some reconstruction results. The reconstructions are achieved by inverting the scattering operator through its adjoint operator. This approach is very common in the literature it is known as migration scheme [39,40]. A complex white Gaussian noise is added to the field data. In particular, a signal to noise ratio (SNR), defined as

(21) $$SNR=\frac{\left||E|\right|}{\left||N|\right|},$$

with ||·|| the norm and N the noise, of 20 dB is considered. Again, our benchmark is the continuous case. Accordingly, $A^{†}$ is obtained by very densely sampling the frequencies and the angles. Instead, when the optimal and suboptimal grids are considered, the data are first interpolated on the above-mentioned dense grid and then $A^{†}$ is applied.

**Figure 8:**
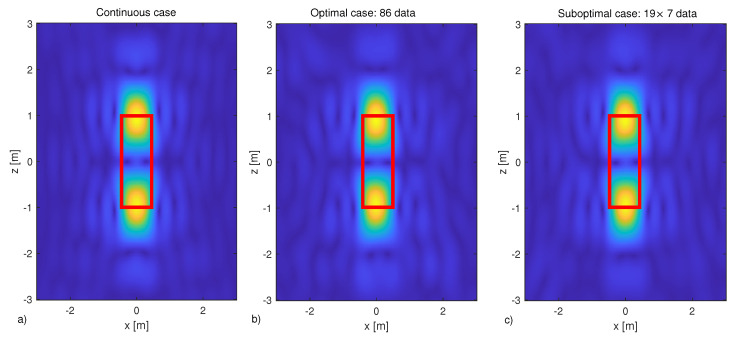
Normalized reconstruction of a 1 m × 2 m rectangular scatterer centered in (0,0) m. The scattering scenario is $ID=\left[-3\lambda_{max},3\lambda_{max}\right]\times \left[-3\lambda_{max},3\lambda_{max}\right]$, $\Omega_{o}=\left[-\frac{\pi }{3},\frac{\pi }{3}\right]rad$ and $\Omega_{k}=\left[k_{0},\frac{3}{2}k_{0}\right]$. In panel (**a**), the reconstruction is obtained by taking continuously the measurements, in panels (**b**,**c**) measurements are collected in optimal and suboptimal grids, respectively. The red lines border the actual scatterer shape. SNR = 20 dB.

**Figure 9:**
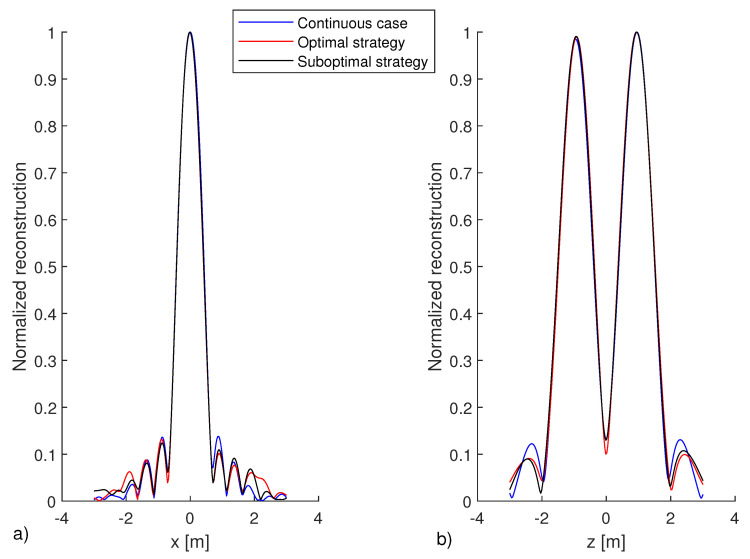
Cut views along *x* and *z* axis passing by (0,1), i.e., where the maximum of the normalized reconstructions reported in Figure 8 are located. SNR = 20 dB.

As a scattering target, dielectric square object 1m×2m in size and centered at (0,0)m is considered. Figure 8 shows the corresponding reconstruction results: panel (a) refers to the continuous case, in panel (b) and (c), the reconstructions are obtained by collecting the field according to the two proposed sampling schemes, the optimal and suboptimal one, respectively. The corresponding cuts along *x* and *z* axes, passing through the maximum of the reconstruction at (0,1), are reported in Figure 9. As expected, due to the adopted linear inversion, only a qualitative reconstruction of the target is obtained. In fact, only the scatterer’s “discontinuities” along the *z* axes are clearly distinguishable due to the “high-pass” filtering introduced by the reconstruction algorithm, which is typical of the considered measurement configuration according to [9,10]. However, what matters here is that both the proposed sampling strategies allow the obtaining of reconstructions that are practically the same as the one returned by inverting the continuously collected data.

## 6. Conclusions

In this paper, a measurement collection problem has been addressed in the framework of inverse scattering. In particular, an optimal sampling strategy for the case of the field collected with a multi-static and multi-frequency configuration in far zone has been proposed. The latter allows minimizing the number of both frequency and spatial measurements by returning several data close to NDF. Unfortunately, such a strategy could lead to a complicated measurement configuration which requires collecting the data at different spatial positions for each frequency. To simplify the measurement configuration, a suboptimal iterative sampling strategy is implemented which enforces the sampling points to belong to a rectangular grid in the spatial frequency domain. As a result of this procedure, the overall data points (i.e., the couples angle-frequency) actually increases but the number of different angles and frequencies reduce and lead to a measurement configuration that is more practical to be implemented.

However, note that the optimal sampling grid could be exploited as starting point of all iterative procedures which implement a sensor selection problem [17,18,19]. Finally, although the results are peculiar for the considered configuration, both the approach can be extended to all scattering configurations for example by also introducing the view diversity and/or by collecting the data in near zone. In the latter case, the problem is a little bit more challenging because it needs to cope with the spatially varying bandwidth of the scattered field [13,14,15].

## Data Availability

No new data were created or analyzed in this study. Data sharing is not applicable to this article.
